# Pangenome graph layout by Path-Guided Stochastic Gradient Descent

**DOI:** 10.1093/bioinformatics/btae363

**Published:** 2024-07-03

**Authors:** Simon Heumos, Andrea Guarracino, Jan-Niklas M Schmelzle, Jiajie Li, Zhiru Zhang, Jörg Hagmann, Sven Nahnsen, Pjotr Prins, Erik Garrison

**Affiliations:** Quantitative Biology Center (QBiC), University of Tübingen, 72076 Tübingen, Germany; Biomedical Data Science, Department of Computer Science, University of Tübingen, 72076 Tübingen, Germany; M3 Research Center, University Hospital Tübingen, 72076 Tübingen, Germany; Institute for Bioinformatics and Medical Informatics (IBMI), University of Tübingen, 72076 Tübingen, Germany; Department of Genetics, Genomics and Informatics, University of Tennessee Health Science Center, Memphis, TN 38163, United States; Genomics Research Centre, Human Technopole, 20157 Milan, Italy; Department of Computer Engineering, School of Computation, Information and Technology (CIT), Technical University of Munich, 80333 Munich, Germany; School of Electrical and Computer Engineering, Cornell University, Ithaca, NY 14853, United States; School of Electrical and Computer Engineering, Cornell University, Ithaca, NY 14853, United States; School of Electrical and Computer Engineering, Cornell University, Ithaca, NY 14853, United States; Computomics GmbH, 72072 Tübingen, Germany; Quantitative Biology Center (QBiC), University of Tübingen, 72076 Tübingen, Germany; Biomedical Data Science, Department of Computer Science, University of Tübingen, 72076 Tübingen, Germany; M3 Research Center, University Hospital Tübingen, 72076 Tübingen, Germany; Institute for Bioinformatics and Medical Informatics (IBMI), University of Tübingen, 72076 Tübingen, Germany; Department of Genetics, Genomics and Informatics, University of Tennessee Health Science Center, Memphis, TN 38163, United States; Department of Genetics, Genomics and Informatics, University of Tennessee Health Science Center, Memphis, TN 38163, United States

## Abstract

**Motivation:**

The increasing availability of complete genomes demands for models to study genomic variability within entire populations. Pangenome graphs capture the full genomic similarity and diversity between multiple genomes. In order to understand them, we need to see them. For visualization, we need a human-readable graph layout: a graph embedding in low (e.g. two) dimensional depictions. Due to a pangenome graph’s potential excessive size, this is a significant challenge.

**Results:**

In response, we introduce a novel graph layout algorithm: the Path-Guided Stochastic Gradient Descent (PG-SGD). PG-SGD uses the genomes, represented in the pangenome graph as paths, as an embedded positional system to sample genomic distances between pairs of nodes. This avoids the quadratic cost seen in previous versions of graph drawing by SGD. We show that our implementation efficiently computes the low-dimensional layouts of gigabase-scale pangenome graphs, unveiling their biological features.

**Availability and implementation:**

We integrated PG-SGD in *ODGI* which is released as free software under the MIT open source license. Source code is available at https://github.com/pangenome/odgi.

## 1 Introduction

Reference genomes are widely used in genomics, serving as a foundation for a variety of analyses, including gene annotation, read mapping, and variant detection (Singh *et al.* 2022). However, this linear model is becoming obsolete given the accessibility to hundreds or even thousands of high-quality genomes. A single genome cannot fully represent the genetic diversity of any species, resulting in reference bias (Ballouz *et al.* 2019). In contrast, a pangenome models the entire set of genomic elements of a given population (Tettelin *et al.* 2008, Computational Pan-Genomics Consortium 2018, Eizenga *et al.* 2020, Sherman and Salzberg 2020). Pangenomes can be represented as a sequence graph incorporating sequences as nodes and their relationships as edges (Hein 1989). In the variation graph model (Garrison *et al.* 2018), genomes are encoded as paths traversing the nodes in the graph.

A graph layout is the arrangement of nodes and edges in an *N*-dimensional space. Graph layout algorithms aim to find optimal node coordinates in order to minimize overlapping nodes or edges, reduce edge crossings, and promote an intuitive understanding of the graph. One popular approach is force-directed graph drawing (Cheong and Si 2022) which uses physical simulation to produce esthetic layouts. The classical approach combines repulsive forces on all vertices and attractive forces on adjacent vertices. This is prone to get stuck in local minima, but multi-layer strategies such as the Fast Multipole Multilevel Method (FM^3^) (Hachul and Jünger 2005) or Stochastic Gradient Descent (SGD) implementations alleviate such a problem (Zheng *et al.* 2019). SGD uses the gradient of its individual terms to approximate the gradient of a sum of functions.

A *pangenome* graph layout can provide a human-readable visualization of genetic variation between multiple genomes. However, Zheng *et al.* (2019)’s algorithm has a quadratic up front cost in the number of nodes to find pairwise distances to guide the layout, making it impossible to apply to pangenome graphs with millions of nodes. Also, existing generic graph layout approaches ignore the biological information inherent in pangenome graphs. One such bioinformatics tool is *BandageNG*, the current state of the art for genome graph visualization. It uses FM^3^ which only considers the nodes and edges of a graph.

In practice, MultiDimensional Scaling (MDS) is applied to minimize the difference between the visual distance and theoretical graph distance. This can be accomplished by using pairwise node distances to minimize an energy function. Since pangenome graphs represent genomes as paths in the graph, a reasonable distance metric would be the nucleotide distance between a pair of nodes traversed by the same path. Such path sampling would overcome the quadratic costs of previous versions of graph drawing by SGD.

Typically, force-directed layouts are hard to compute (Wang *et al.* 2014). Although, *BandageNG* applies FM^3^ for layout generation, its parallelism is bound by the number of connected graph components. Alternatively, the lock-free HOGWILD! method offers a highly parallelizable and thus scalable SGD approach that can be applied when the optimization problem is sparse (Recht *et al.* 2011).

Here, we present a new pangenome graph layout algorithm which applies a Path-Guided SGD (PG-SGD) to use the paths as an embedded positional system to find distances between nodes, moving pairs of nodes in parallel with a modified HOGWILD! strategy. The algorithm computes the pangenome graph layout that best reflects the nucleotide sequences in the graph. To our knowledge, no generic graph layout algorithm takes into account such path encoded biological information when computing a graph’s layout.

PG-SGD can be extended in any number of dimensions. In the ODGI toolkit (Guarracino *et al.* 2022), we provide implementations for 1D and 2D layouts. These algorithms have already been successfully applied to construct and visualize large-scale pangenome graphs of the Human Pangenome Reference Consortium (HPRC) (Guarracino *et al.* 2023, Liao *et al.* 2023). In addition, we show that PG-SGD is almost an order of magnitude faster than *BandageNG*.

## 2 Algorithm

While PG-SGD is inspired by Zheng *et al.* (2019), we designed the algorithm to work on the variation graph model (Definition 2.1).

Definition 2.1. Variation graphs are a mathematical formalism to represent pangenome graphs (Garrison 2019). In the variation graph G=(V,E,P), nodes (or vertices) $V=v_{1}\ldots v_{\left|V\right|}$ contain nucleotide sequences. Each node *v_i_* has a unique identifier *i* and an implicit reverse complement $\bar{v_{i}}$. The node strand *o* represents the node orientation. Edges $E=e_{1}\ldots e_{\left|E\right|}$ connect ordered pairs of node strands ($e_{i}=(o_{a},o_{b})$), defining the graph topology. Paths $P=p_{1}\ldots p_{\left|P\right|}$ are series of connected steps *s_i_* that refer to node strands in the graph ($p_{i}=s_{1}\ldots s_{\left|p_{i}\right|}$); the paths represent the genomes embedded in the graph.

**Figure 1.**
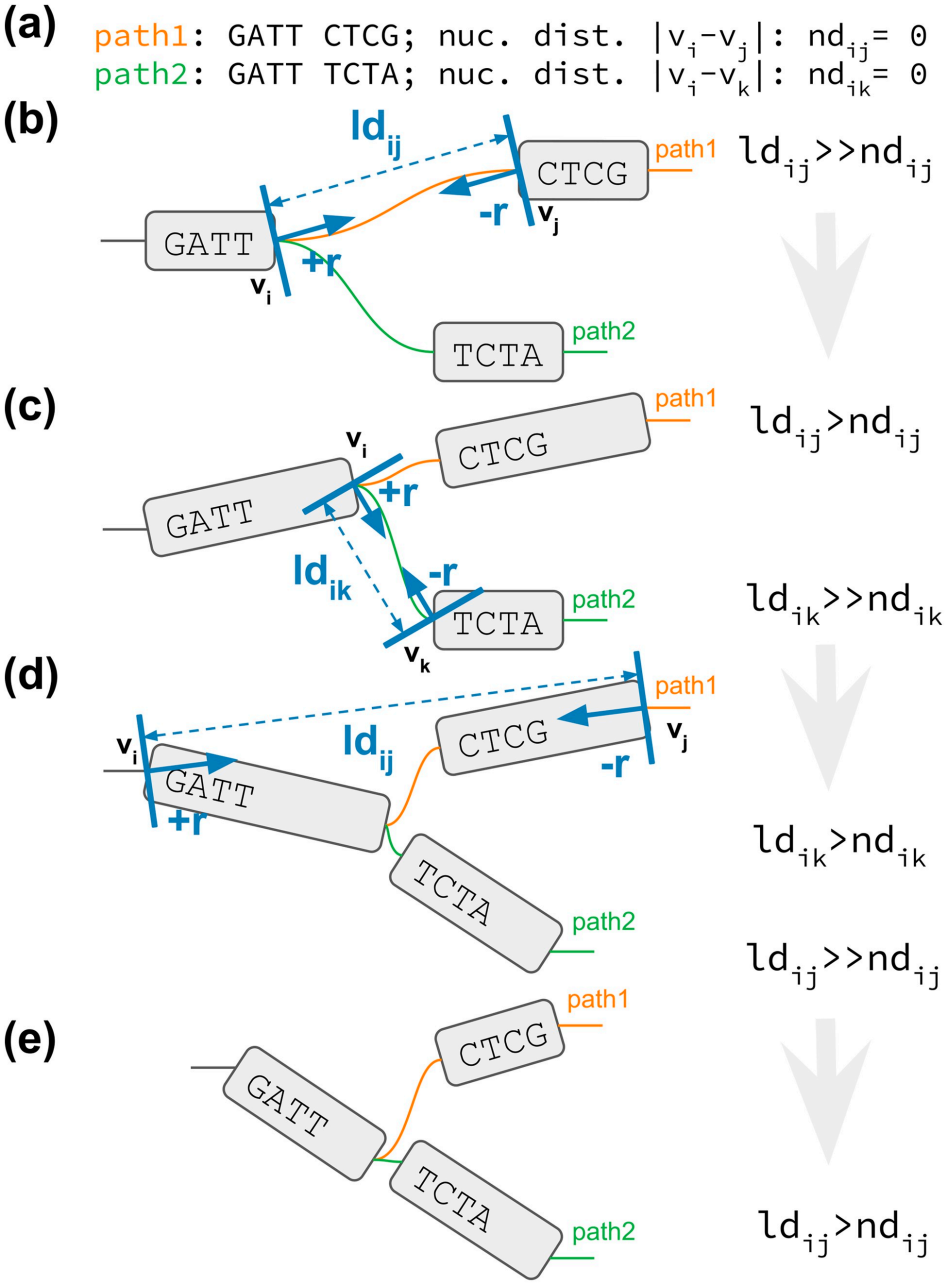
2D PG-SGD update operation sketches. (a) The path information of the graph. *path1* and *path2* both visit the same first node. Then their sequence diverges and they visit distinct nodes. (b–e) *v_i_*/*v_j_* or *v_i_*/*v_k_* is the current pair of nodes to update. *ld_ij_*/*ld_ik_* is the current layout distance. r,−r is the current size of the update. (b) Initial graph layout highlighting the future update of the two nodes of *path1*. (c) The graph layout after the first update. The nodes appear longer now, because we updated at the end of the nodes. Highlighted is the future update of the two nodes of *path2*. (d) The graph layout after the second update. Highlighted is the future update of the two nodes of *path1*. (e) Final graph layout after three updates using the 2D PG-SGD.

We report PG-SGD’s pseudocode in Algorithm 1 and its schematic in Fig. 1. In brief, the algorithm moves one pair of nodes $\left(v_{i},v_{j}\right)$ at a time, minimizing the difference between the layout distance *ld_ij_* of the two nodes and the nucleotide distance *nd_ij_* of the same nodes as calculated along a path that traverses them. In the 2D layouts, nodes have two ends. When moving a pair of nodes, we actually move one end of each node. For clarification, an example is given in Fig. 1. *v_i_* is the node associated with the step *s_i_* sampled uniformly from all the steps in P. *v_j_* is the node associated with the step *s_j_* sampled from the same path of *s_i_* by drawing a uniform or a Zipfian distribution (Zipf 1932). The difference between *nd_ij_* and *ld_ij_* guides the update of the node coordinates in the layout. The magnitude *r* of the update depends on the learning rate *μ*. The number of iterations steers the annealing step size *η* which determines the learning rate *μ*. A large *η* in the first iterations leads to a globally linear (in 1D) or planar (in 2D) layout. By decreasing *η*, the layout adjustments become more localized, ensuring that the nodes are positioned to best reflect the nucleotide distances in the paths (i.e. in the genomes).

**Algorithm 1:** Pseudocode of PG-SGD in 1D.

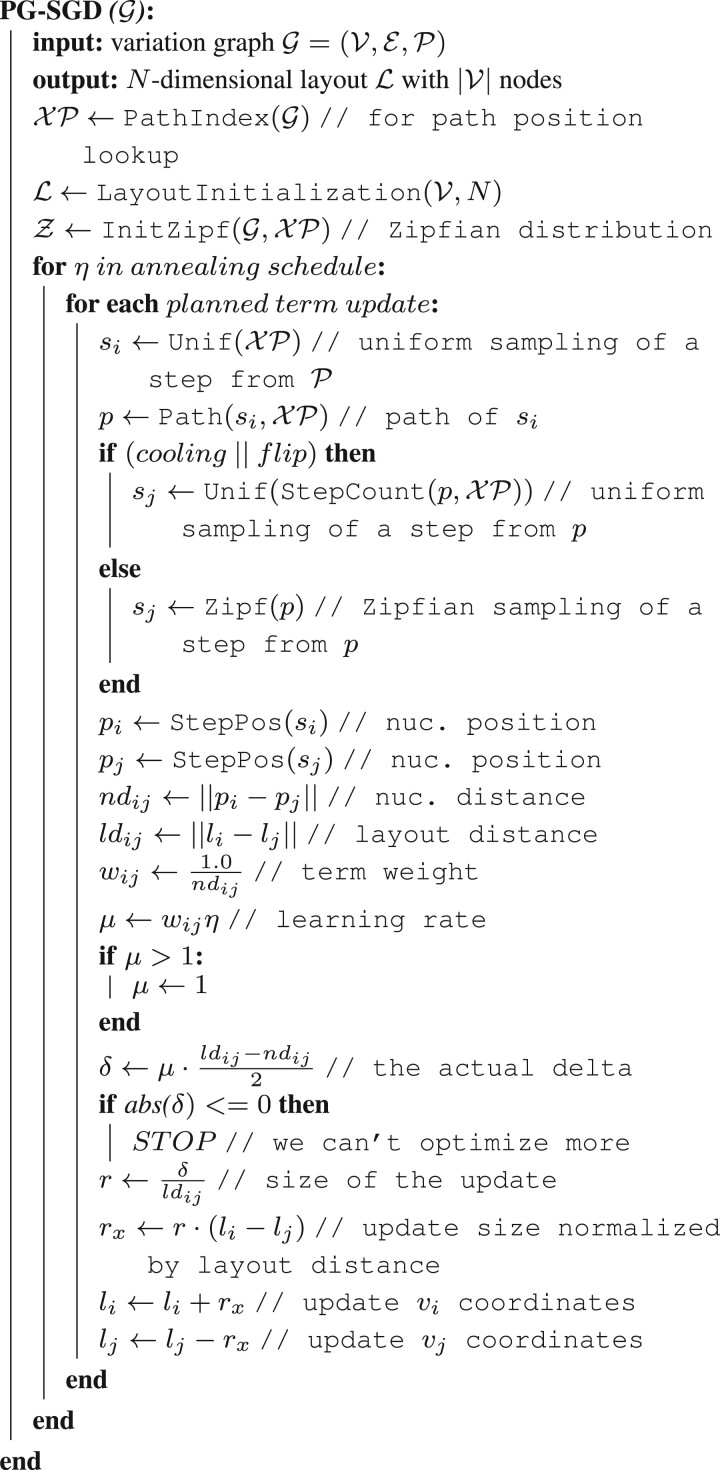

Originating from empirical inspection of word frequency tables, Zipf’s law states that a word with rank *n* occurs 1/n times as the most frequent one. This law is modeled by the Zipf distribution. Sampling *s_j_* from a Zipf distribution fixed in the *s_i’_*s path position space increases the possibility to draw a nucleotide position close to *s_i_*. So there is a high chance to use small nucleotide distances *nd_ij_* to refine the layout of nodes comprising a few base pairs. The Zipf distribution is also long-tailed, with many occurrences of low frequency events. However, extremely long-range correlations might not be captured sufficiently, resulting in collapsed layouts for structures that are otherwise linear. To provide balance between global and local layout updates, in half of the updates (*flip* flag in Algorithm 1), the *s_j_* is sampled uniformly instead from a Zipf distribution, with uniform sampling being more favorable for global updates. Furthermore, to enhance local linearity (in 1D) or planarity (in 2D) of the graph layout, a *cooling* phase skews the Zipfian distribution after half of iterations have been completed. This increases the likelihood of sampling smaller nucleotide distances for the layout updates.

## 3 Implementation

We implemented PG-SGD in ODGI (Guarracino *et al.* 2022): the 1D version can be found in *odgi sort* and the 2D version in *odgi layout*. To efficiently retrieve path nucleotide positions, we implemented a path index. This index is a strict subset of the XG index (Garrison *et al.* 2018) where we avoid to use succinct SDSL data structures (Gog *et al.* 2014). Instead, we rely on bit-compressed integer vectors, enabling efficient retrieval of path nucleotide positions to quickly compute nucleotide distances without having to store all pairwise distances between nodes in memory. This approach ensures to scale on large pangenome graphs representing thousands of whole genomes.

Graph layout initialization can significantly influence the quality of the final layout. In the 1D implementation, by default, nodes are placed in the same order as they appear in the input graph, although we also provide support for random layout initialization. In 2D, we offer several layout initialization techniques. One approach places nodes in the first layout dimension according to their order in the input graph, adding either uniform or Gaussian noise in the second dimension. Another strategy arranges nodes along a Hilbert curve, an approach that often favors the creation of planar final layouts. We also support fixing node positions to keep nodes in the same order as they are in a selected path, such as a reference genome. This feature allows us to build reference-focused graph layouts (Supplementary Fig. S1d).

Our implementation is multithreaded and uses shared memory for storing the layout in a vector, according to the HOGWILD! strategy (Recht *et al.* 2011). Threads perform layout updates without any locking for additional speed up. This approach is feasible since pangenome graphs are typically sparse (Guarracino *et al.* 2022), with low average node degree. As a result, the updates only modify small parts of the entire layout. While the HOGWILD! SGD algorithm writes the layout updates to a shared non-atomic double vector, PG-SGD stores node coordinates in a vector of atomic doubles. This vector prevents any potential memory overwrites. Our tests revealed basically no performance loss with respect to the non-atomic counterpart.

## 4 Results

### 4.1 Performance

We apply the 2D PG-SGD to the human pangenome (Liao *et al.* 2023) from the HPRC to show the scalability of the algorithm. Experiments were conducted on a cluster with 24 Regular nodes (32 cores/64 threads with two AMD EPYC 7343 processors with 512 GB RAM) and 4 HighMem nodes (64 cores/128 threads with two AMD EPYC 7513 processors with 2048 GB RAM). We downloaded pangenome graphs for each autosome (24 in total) and for the mitochondrial DNA. Each graph represents 90 whole human haplotypes: 44 diploid individuals plus the GRCh38 (Schneider *et al.* 2017) and CHM13 (Nurk *et al.* 2021) haploid human references (see Supplementary Table S1 for graph statistics). When applied to these pangenome graphs using one Regular node for each calculation, *odgi layout’*s 2D PG-SGD implementation obtains the graph layouts in 50 min on average, with the highest run time observed being chromosome 16 (Supplementary Table S1). This is expected since chromosome 16 has one of the highest levels of segmentally duplicated sequence among the human autosomes (Martin *et al.* 2004). Repetitive sequences lead to graph nodes with a very high number of path steps, which are computationally expensive to work with (Guarracino *et al.* 2022). Memory consumption is 29.66 GB of RAM on average, with the memory peak again occurring with chromosome 16, due to the path index building phase. Given its scalability, we applied 2D PG-SGD to the full graph with all chromosomes together using a HighMem node (Supplementary Table S1). To compare, *BandageNG* (https://github.com/asl/BandageNG, last accessed July 2023), the current state of the art for graph visualization, was used to calculate a 2D layout of each of the HPRC pangenome graphs. For a fair comparison, we did not rely on *BandageNG*’s interactive GUI application, but we executed *BandageNG layout*, which directly emits a 2D graph layout similar to *odgi layout*. *BandageNG* was not able to produce a layout for the full graph within 7 days, hitting the wall clock time limit of the cluster. On average, PG-SGD is ∼8× faster than *BandageNG* while using ∼2× less memory.

### 4.2 Pangenome graph layouts reveal biological features

**Figure 2.**
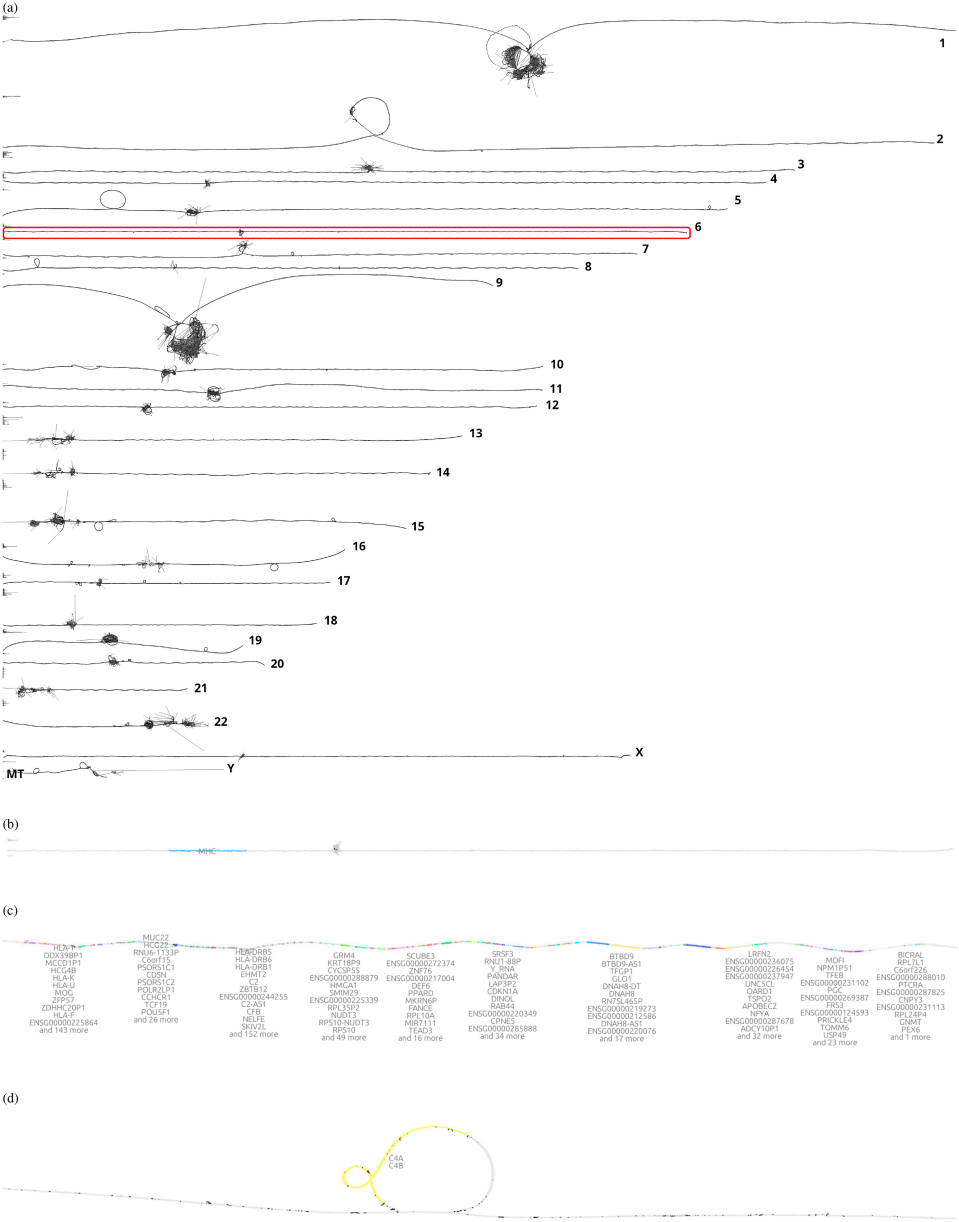
2D visualizations of all chromosomes of the Human Pangenome Reference Consortium (HPRC) 90 haplotypes pangenome graph, chromosome 6, the major histocompatibility complex (MHC), and the complement component 4 (C4). (a) *odgi draw* layout of the HPRC pangenome graph 90 haplotypes. Displayed are all 24 autosomes and the mitochondrial chromosome. A red rectangle highlights chromosome 6 which is shown in the subfigure below. (b) *gfaestus* screenshot of the chromosome 6 layout. Colored in blue is the MHC. The hairball in the middle is the centromere. The black structures in the centromere are edges. (c) *gfaestus* screenshot of the MHC. All MHC genes are color annotated and the names of the genes appear as a text overlay. (d) *gfaestus* screenshot of the region around C4, specifically color highlighting genes C4A and C4B. The black lines are the edges of the graph.

Graph visualization is essential for understanding pangenome graphs and the genome variation they represent. We show how 2D PG-SGD allows us gaining insight into biological data by looking at the graph layout structure. In Fig. 2a, the chromosomes of the HPRC graph show the large-scale structural variations in the centromeres. Focusing on the major histocompatibility complex (MHC) of chromosome 6 (Fig. 2b), the 2D layout reveals the positions and diversity of all MHC genes (Fig. 2c). In Fig. 2d, the C4A and C4B genes are highlighted. Complementary, we provide various 1D visualizations in Supplementary Fig. S1.

## 5 Discussion

We presented PG-SGD, the first layout algorithm for pangenome graphs that leverages the biological information available within the genomes represented in the graph. Other generic graph layout algorithms, such as the one offered by *BandageNG*, ignore this additional information. Our implementation efficiently computes the layout of pangenome graphs representing thousands of whole genomes.

Graph visualization is key for understanding genome variations and the layouts produced by PG-SGD offer an unprecedented high-level perspective on pangenome variation. We implemented PG-SGD to generate layouts in 1D and 2D. These graph projections have already been employed in constructing and analyzing the first draft human pangenome reference (Liao *et al.* 2023), as well as in the discovery of heterologous recombination of human acrocentric chromosomes (Guarracino *et al.* 2023). Furthermore, they are applied in the creation and analysis of pangenome graphs for any species (Guarracino *et al.* 2022, Garrison *et al.* 2023). Of note, there still remains a gap in interactive and scalable solutions that merge layouts of large pangenome graphs with annotation. Our algorithm will underpin new pangenome graph browsers for studying graph layouts and the genome variation they represent (https://github.com/chfi/waragraph, last accessed July 2023).

The performance analysis shows that our 2D implementation outperforms *BandageNG* when handling large, complex pangenome graphs. While *BandageNG* was not able to deliver a layout of the whole HPRC graph within 1 week, our 2D PG-SGD calculated one within one day. There are some possible optimization approaches for future work to further improve the performance of PG-SGD, making it possible for interactive use. The data structure could be optimized to improve cache performance. Moreover, the high-degree of parallelism could be further exploited by using a GPU. In *BandageNG*, one cannot select the number of threads for the calculations. They are automatically chosen by the number of connected components of the graph to draw. This limits its parallelism and leads to an unbalanced workload. Since *BandageNG* was primarily designed for assembly graphs, one may have to adjust its parameters dependent on the input graph, in order to boost the layout generation or to adjust the highlighting of desired graph features.

The classical force model of state of the art generic graph algorithms, such as FM^3^-based ones, places nodes according to their attractive and repulsive forces. This force can be seen as equivalent to how our 2D PG-SGD moves the nodes’ ends in 2D: If the nucleotide distance of the randomly chosen path steps is smaller than the layout distance of the nodes’ ends, we move them closer together (“attractive force”), else we move them further away (“repulsive force”). However, the key difference here is that this approach is path-guided: paths represent biological sequences in pangenome graphs, so it is as if PG-SDG considers a “biological force” for placing the graph nodes. Theoretically, it would be possible to combine our approach with a force-directed one. Combining both methods, we might get the best of both worlds: multi-threadable PG-SGD iteratively applied to different graph layout-levels. We can imagine that such an approach can lead to a further speedup when calculating the layout. However, for generic graphs, this would only work if path information for each node could be added: we would replace the classical physical simulation approach with our path-guided method. If such information is not available, one could randomly cover the graph with paths. This function is already provided in *odgi cover*. However, this is an NP-hard problem and our preliminary solutions proved ineffective.

With assembly graphs we face the same problem: they usually do not carry path information during each assembly step. One could map the initial assembly reads back against the assembly graph in order to build paths through the graph. This would allow us to obtain a layout using PG-SGD.

PG-SGD can be extended to any number of dimensions. It can be seen as a graph embedding algorithm that converts high-dimensional, sparse pangenome graphs into low-dimensional, dense, and continuous vector spaces, while preserving its biologically relevant information. This enables the application of machine learning algorithms that use the graph layout for variant detection and classification. Our future research involves leveraging these graph projections to detect structural variants and to identify and correct assembly errors. Moreover, we are considering extending the algorithm to RNA and protein sequences to support pantranscriptome graphs (Sibbesen *et al.* 2023) and panproteome graphs (Dabbaghie *et al.* 2023), respectively.

## Supplementary Material

btae363_Supplementary_Data

## Data Availability

Software versions, code, and links to data used to prepare this manuscript can be found at https://github.com/pangenome/sorting-paper. Animations of the algorithm are deposited at https://doi.org/10.5281/zenodo.8288999.

## References

[btae363-B1] Ballouz S , Dobin A, Gillis JA et al Is it time to change the reference genome? Genome Biol 2019;20:159. 31399121 10.1186/s13059-019-1774-4 PMC6688217

[btae363-B2] Cheong S-H , Si Y-W. Force-directed algorithms for schematic drawings and placement: a survey. Inf Vis 2019;9:65–91.

[btae363-B3] Computational Pan-Genomics Consortium. Computational pan-genomics: status, promises and challenges. Brief Bioinform 2018;19:118–35. 27769991 10.1093/bib/bbw089 PMC5862344

[btae363-B4] Dabbaghie F , Srikakulam SK, Marschall T et al PanPA: generation and alignment of panproteome graphs. Bioinformatics 2023;3:vbad167. 10.1093/bioadv/vbad167 PMC10748787 38145107

[btae363-B5] Eizenga JM , Novak AM, Sibbesen JA et al Pangenome graphs. Annu Rev Genomics Hum Genet 2020;21:139–62. 32453966 10.1146/annurev-genom-120219-080406 PMC8006571

[btae363-B6] Garrison E. Graphical pangenomics. Apollo – University of Cambridge Repository 2019.

[btae363-B7] Garrison E , Sirén J, Novak AM et al Variation graph toolkit improves read mapping by representing genetic variation in the reference. Nat Biotechnol 2018;36:875–9. 30125266 10.1038/nbt.4227 PMC6126949

[btae363-B8] Garrison E , Guarracino A, Heumos S et al Building pangenome graphs. bioRxiv 2023.

[btae363-B9] Gog S , Beller T, Moffat A et al From theory to practice: plug and play with succinct data structures. In: *13th International Symposium on Experimental Algorithms, (SEA 2014). Springer International Publishing* 2014, 326–37.

[btae363-B10] Guarracino A , Heumos S, Nahnsen S et al ODGI: understanding pangenome graphs. Bioinformatics 2022;38:3319–26. 35552372 10.1093/bioinformatics/btac308 PMC9237687

[btae363-B11] Guarracino A , Buonaiuto S, de Lima LG et al Recombination between heterologous human acrocentric chromosomes. Nature 2023;617:335–43. 37165241 10.1038/s41586-023-05976-y PMC10172130

[btae363-B12] Hachul S , Jünger M. Large-graph layout with the fast multipole multilevel method. Working Paper, Universität zu Köln, 2005.

[btae363-B13] Hein J. A new method that simultaneously aligns and reconstructs ancestral sequences for any number of homologous sequences, when the phylogeny is given. Mol Biol Evol 1989;6:649–68. 2488477 10.1093/oxfordjournals.molbev.a040577

[btae363-B14] Liao W-W , Asri M, Ebler J et al A draft human pangenome reference. Nature 2023;617:312–24. 37165242 10.1038/s41586-023-05896-x PMC10172123

[btae363-B15] Martin J , Han C, Gordon LA et al The sequence and analysis of duplication-rich human chromosome 16. Nature 2004;432:988–94. 15616553 10.1038/nature03187

[btae363-B16] Nurk S , Koren S, Rhie A et al The complete sequence of a human genome. Science 2022;376:44–53. 35357919 10.1126/science.abj6987 PMC9186530

[btae363-B17] Recht B , Re C, Wright S et al Hogwild!: a lock-free approach to parallelizing stochastic gradient descent. Advances in Neural Information Processing Systems; 24. Curran Associates, Inc., 2011.

[btae363-B18] Schneider VA , Graves-Lindsay T, Howe K et al Evaluation of GRCh38 and de novo haploid genome assemblies demonstrates the enduring quality of the reference assembly. Genome Res 2017;27:849–64. 28396521 10.1101/gr.213611.116 PMC5411779

[btae363-B19] Sherman RM , Salzberg SL. Pan-genomics in the human genome era. Nat Rev Genet 2020;21:243–54. 32034321 10.1038/s41576-020-0210-7 PMC7752153

[btae363-B20] Sibbesen JA , Eizenga JM, Novak AM et al Haplotype-aware pantranscriptome analyses using spliced pangenome graphs. Nat Methods 2023;20:239–47. 36646895 10.1038/s41592-022-01731-9

[btae363-B21] Singh V , Pandey S, Bhardwaj A et al From the reference human genome to human pangenome: premise, promise and challenge. Front Genet 2022;13:1042550. 36437921 10.3389/fgene.2022.1042550 PMC9684177

[btae363-B22] Tettelin H , Riley D, Cattuto C et al Comparative genomics: the bacterial pan-genome. Curr Opin Microbiol 2008;11:472–7. 19086349 10.1016/j.mib.2008.09.006

[btae363-B23] Wang L , Wang X, Wang Q et al Research on force-directed algorithm optimization methods. In: *Proceedings of the 2014 International Conference on e-Education, e-Business and Information Management (ICEEIM 2014)*, *Shanghai, China*. Atlantis Press 2014.

[btae363-B24] Zheng JX , Pawar S, Goodman DFM et al Graph drawing by stochastic gradient descent. IEEE Trans Vis Comput Graph 2019;25:2738–48. 30047888 10.1109/TVCG.2018.2859997

[btae363-B25] Zipf GK. Selected Studies of the Principle of Relative Frequency in Language. Cambridge, MA/London, England: Harvard University Press, 1932.

