# Supplementary material for: Pangenome graph layout by Path-Guided Stochastic Gradient Descent
Source: Bioinformatics. 2024 Jul 3;40(7):btae363. doi: 10.1093/bioinformatics/btae363 (PMC11227364; doi:10.1093/bioinformatics/btae363)
Supplement: btae363_Supplementary_Data [file btae363_supplementary_data.pdf]

Table S1: Performance evaluation of computing a 2D layout of all chromosomal HPRC pangenome graphs. From GFA to the actual layout. **comps:** Number of weakly connected components. **pg-sgd:** *odgi layout* 2D PG-SGD implementation. **bng:** *BandageNG layout* implementation. **32T:** Number of threads: 32. **64T:** Number of threads: 64. **BandageNG* did not finish within the job wall clock time limit of 7 days. Therefore, no layout was produced.

							**time in minutes**				**memory in gigabytes**			
							32T		64T		32T		64T	
**name**	**len**	**nodes**	**edges**	**paths**	**steps**	**comps**	**pg − sgd**	**bng**	**pg − sgd**	**bng**	**pg − sgd**	**bng**	**pg − sgd**	**bng**
chr1	1.12e+09	1.11e+07	1.54e+07	2.26e+03	6.01e+08	63	110	1439	**68**	1427	**55.73**	149.91	56.00	195.33
chr2	3.47e+08	6.68e+06	9.27e+06	1.65e+03	3.89e+08	15	67	576	**47**	521	37.31	81.97	**37.29**	81.97
chr3	4.06e+08	6.20e+06	8.62e+06	1.56e+03	4.55e+08	106	81	473	**52**	481	**41.34**	81.41	41.71	93.83
chr4	2.73e+08	5.91e+06	8.23e+06	1.35e+03	4.97e+08	36	88	422	**56**	423	**44.90**	79.40	45.02	79.48
chr5	3.35e+08	5.39e+06	7.51e+06	1.20e+03	4.04e+08	19	73	349	**46**	375	**35.83**	75.13	36.48	75.10
chr6	2.29e+08	4.70e+06	6.56e+06	1.41e+03	4.03e+08	40	70	270	**46**	271	**36.74**	71.25	37.22	71.26
chr7	2.71e+08	5.17e+06	7.25e+06	1.22e+03	4.10e+08	24	70	328	**46**	346	**37.39**	73.70	37.88	73.81
chr8	1.93e+08	4.26e+06	5.95e+06	8.55e+02	4.29e+08	16	71	224	**47**	233	**37.73**	54.72	38.07	54.70
chr9	1.01e+09	8.80e+06	1.23e+07	8.67e+02	3.31e+08	11	44	931	**38**	957	**31.76**	131.93	31.79	131.96
chr10	2.56e+08	4.50e+06	6.26e+06	8.79e+02	2.72e+08	14	36	256	**32**	260	25.32	67.85	**25.25**	67.87
chr11	2.83e+08	4.73e+06	6.54e+06	6.53e+02	2.38e+08	8	31	277	**28**	286	21.81	68.49	**21.77**	68.54
chr12	2.44e+08	4.10e+06	5.71e+06	7.68e+02	2.54e+08	9	44	210	**27**	206	**23.55**	51.19	23.99	51.22
chr13	3.47e+08	4.34e+06	6.08e+06	2.58e+03	3.12e+08	153	52	242	**34**	237	**27.98**	54.02	28.64	85.85
chr14	2.73e+08	4.15e+06	5.79e+06	1.82e+03	2.62e+08	133	45	222	**28**	222	**23.56**	51.67	24.17	78.13
chr15	5.64e+08	5.20e+06	7.26e+06	2.06e+03	4.02e+08	131	64	347	**35**	334	**35.20**	74.27	35.69	102.97
chr16	3.39e+08	3.91e+06	5.53e+06	1.52e+03	6.91e+08	25	**152**	216	512	244	58.88	**53.00**	61.02	**53.00**
chr17	1.73e+08	2.76e+06	3.93e+06	1.42e+03	3.25e+08	77	50	102	**33**	102	**27.83**	40.68	28.69	49.50
chr18	2.44e+08	2.83e+06	3.98e+06	1.27e+03	3.00e+08	111	44	108	**31**	106	**26.61**	40.80	26.78	45.01
chr19	2.91e+08	3.02e+06	4.21e+06	1.07e+03	2.03e+08	19	31	123	**21**	117	**18.12**	40.14	18.43	40.18
chr20	1.87e+08	2.82e+06	3.97e+06	8.24e+02	2.35e+08	17	35	114	**25**	108	**20.79**	39.02	21.04	39.05
chr21	2.74e+08	2.76e+06	3.88e+06	3.03e+03	2.21e+08	218	33	110	**23**	103	**18.79**	38.07	19.12	46.47
chr22	4.64e+08	3.76e+06	5.22e+06	1.76e+03	2.05e+08	82	32	181	**22**	183	**18.30**	44.73	18.65	45.13
chrX	2.07e+08	3.46e+06	4.89e+06	2.42e+03	2.70e+08	11	41	156	**28**	155	**24.66**	43.05	24.84	43.05
chrY	8.80e+07	3.18e+05	4.41e+05	3.07e+02	1.34e+07	7	2	5	**1**	5	**1.47**	4.65	1.57	4.65
chrM	1.76e+04	1.40e+03	1.89e+03	4.40e+01	4.06e+04	1	**1**	**1**	**1**	**1**	0.21	**0.04**	0.49	**0.04**
all chrs	8.42e+09	1.11e+08	1.55e+08	3.48e+04	8.12e+09	1346	1630	-*	**1020**	-*	**737.15**	-*	738.76	-*

6 Supplement

6.0.1 Performance evaluation

The results of the performance evaluation are given in Table S1.

6.0.2 1D visualizations

The 1D PG-SGD algorithm creates a 1D layout of the nodes of the graph. Theoretically, it is possible that 2 nodes have the same 1D coordinate or overlap. But, in our 1D visualizations, we arrange the nodes from left to right. Therefore, we project the 1D coordinates into a 1D node order: We sort the final layout by graph component, graph position, and node rank.

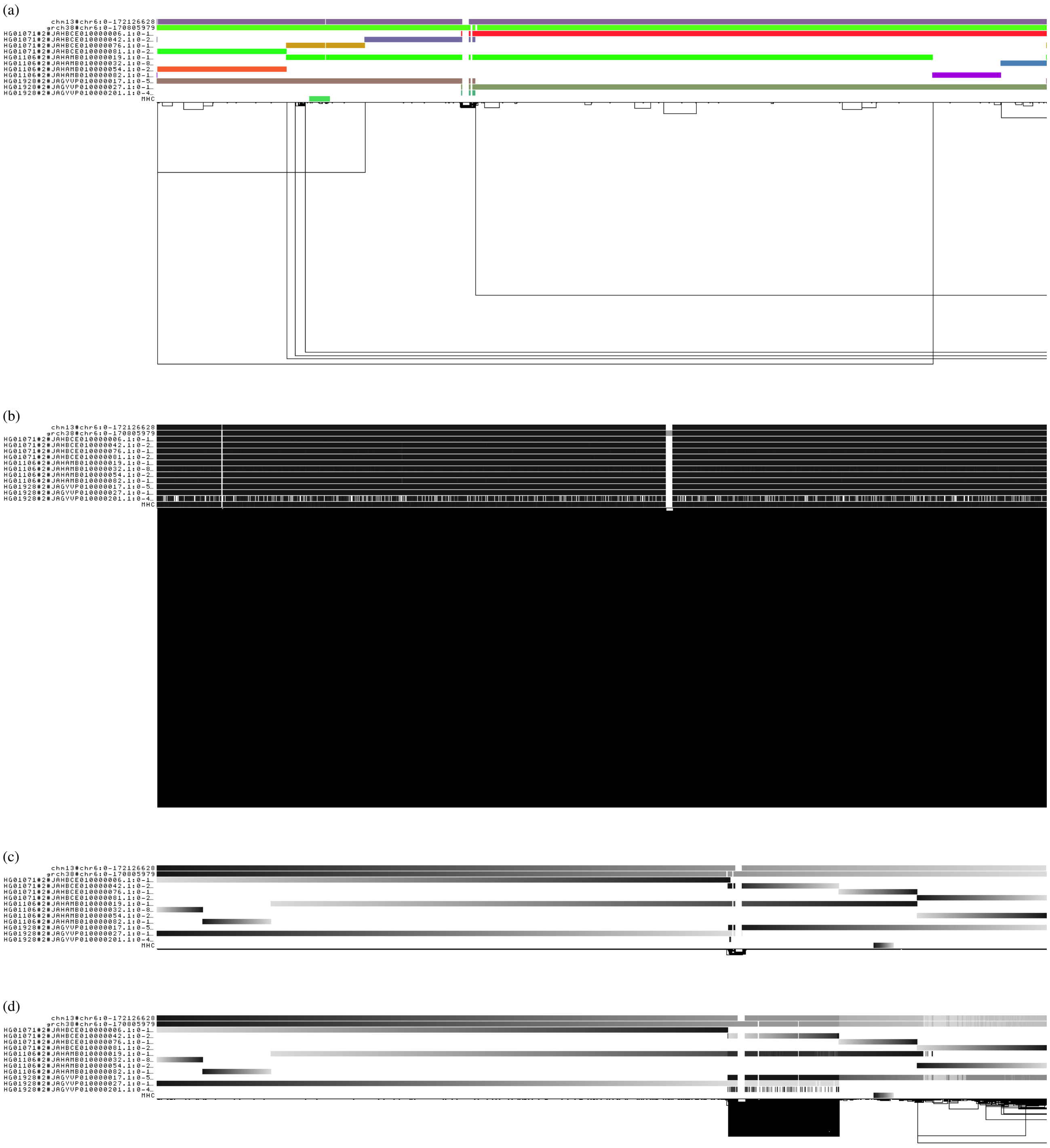

Fig. S1: *odgi viz* 1D visualizations of a 5 haplotypes subgraph of the Human Pangenome Reference Consortium (HPRC) chromosome 6 pangenome graph. The major histocompatibility complex (MHC) sequence was injected as an extra path. Various node arrangements are shown. **a-d** A graphs nodes are arranged from left to right forming the pangenome sequence. The black lines under the paths are the links representing the topology. Path names are left. **(a)** *odgi viz* default modality: The colored bars are the paths versus pangenome sequence in a binary matrix. Shown is the subgraph extracted with *odgi extract*. No 1D layout algorithm was applied here. **b-d** *odgi viz* colored by path position. Light grey corresponds to the beginning of a path, black encodes the end of the path. **(b)** The nodes are arranged randomly in 1D. **(c)** The nodes are arranged applying the 1D PG-SGD algorithm. **(d)** The nodes are arranged applying a 1D reference-guided PG-SGD where the nodes of haplotype HG01071 are fixed and only all the othere ones are movable in 1D. Now all paths of this haplotype are arranged from their lowest to their highest nucleotide position. However,lot's of longer links are now visible compared to the node ordering directly above. This indicates a node order that is globally not optimal.
